# Developing measures to assess constructs from the Inner Setting domain of the Consolidated Framework for Implementation Research

**DOI:** 10.1186/s13012-018-0736-7

**Published:** 2018-03-27

**Authors:** Maria E. Fernandez, Timothy J. Walker, Bryan J. Weiner, William A. Calo, Shuting Liang, Betsy Risendal, Daniela B. Friedman, Shin Ping Tu, Rebecca S. Williams, Sara Jacobs, Alison K. Herrmann, Michelle C. Kegler

**Affiliations:** 1 University of Texas Health Science Center at Houston, Center for Health Promotion and Prevention Research, School of Public Health, 7000 Fannin St, Houston, TX 77030 USA; 2 Department of Global Health, University of Washington, Box 357965, 1510 San Juan Road, Seattle, WA 98195 USA; 3 Department of Public Health Sciences, Penn State College of Medicine, Mail Code CH69 | 500 University Drive, Hershey, PA 17033 USA; 4 Emory Prevention Research Center, Department of Behavioral Sciences and Health Education, Rollins School of Public Health, Emory University, 1518 Clifton Road NE, Atlanta, GA 30033 USA; 5 Department of Community and Behavioral Health, Colorado School of Public Health, University of Colorado Comprehensive Cancer Center, 13001 E. 17th Place, MSF538, Aurora, CO 80045 USA; 6 Department of Health Promotion, Education, and Behavior and the Statewide Cancer Prevention and Control Program, Arnold School of Public Health, University of South Carolina, 915 Greene Street, Columbia, SC 29208 USA; 7 Department of Internal Medicine, University of California Davis, Suite 2400 , 4150 V Street, Sacramento, CA 95817 USA; 8 Center for Health Promotion and Disease Prevention, Lineberger Comprehensive Cancer Center, University of North Carolina at Chapel Hill, CB 7424, Chapel Hill, NC 27599 USA; 9 Public Health Research Division, RTI International, 3040 East Cornwallis Road, Research Triangle Park, Durham, NC 27709-2194 USA; 10 UCLA Kaiser Permanente Center for Health Equity, Fielding School of Public Health and Jonsson Comprehensive Cancer Center, 650 Charles E. Young Dr. S., A2-125 CHS, Box 690015, Los Angeles, CA 90095-6900 USA

**Keywords:** Consolidated Framework for Implementation Research, CFIR, Inner Setting, Measurement of implementation, Implementation science, Colorectal cancer screening implementation

## Abstract

**Background:**

Scientists and practitioners alike need reliable, valid measures of contextual factors that influence implementation. Yet, few existing measures demonstrate reliability or validity. To meet this need, we developed and assessed the psychometric properties of measures of several constructs within the *Inner Setting* domain of the Consolidated Framework for Implementation Research (CFIR).

**Methods:**

We searched the literature for existing measures for the 7 Inner Setting domain constructs (*Culture Overall*, *Culture Stress*, *Culture Effort*, *Implementation Climate*, *Learning Climate*, *Leadership Engagement*, *and Available Resources*). We adapted items for the healthcare context, pilot-tested the adapted measures in 4 Federally Qualified Health Centers (FQHCs), and implemented the revised measures in 78 FQHCs in the 7 states (*N* = 327 respondents) with a focus on colorectal cancer (CRC) screening practices. To psychometrically assess our measures, we conducted confirmatory factor analysis models (CFA; structural validity), assessed inter-item consistency (reliability), computed scale correlations (discriminant validity), and calculated inter-rater reliability and agreement (organization-level construct reliability and validity).

**Results:**

CFAs for most constructs exhibited good model fit (CFI > 0.90, TLI > 0.90, SRMR < 0.08, RMSEA < 0.08), with almost all factor loadings exceeding 0.40. Scale reliabilities ranged from good (0.7 ≤ *α* < 0.9) to excellent (*α* ≥ 0.9). Scale correlations fell below 0.90, indicating discriminant validity. Inter-rater reliability and agreement were sufficiently high to justify measuring constructs at the clinic-level.

**Conclusions:**

Our findings provide psychometric evidence in support of the CFIR Inner Setting measures. Our findings also suggest the Inner Setting measures from individuals can be aggregated to represent the clinic-level. Measurement of the Inner Setting constructs can be useful in better understanding and predicting implementation in FQHCs and can be used to identify targets of strategies to accelerate and enhance implementation efforts in FQHCs.

## Background

Translating the most recent evidence of what works in disease prevention, diagnosis, and treatment into routine practice in a timely fashion has been a significant challenge for both researchers and practitioners [1–4]. This challenge can be even greater for community clinics such as Federally Qualified Health Centers (FQHC) that struggle to meet evolving needs of their patients and demands of their organizations and funders. Despite these challenges, it is clear that to improve the quality and effectiveness of primary care, it is essential to accelerate and improve the implementation of evidence-based approaches (EBAs). There are many models and frameworks, such as the Consolidated Framework for Implementation Research (CFIR), that describe contextual factors associated with implementation, yet scientists’ ability to accurately measure and intervene upon those factors has been limited.

To advance the field of implementation science and to enable better understanding of factors influencing implementation, accurate and valid measurement is crucial. Nevertheless, systematic reviews reveal that many available measures of implementation context, process, and outcomes lack reliability or validity [5–8]. An urgent need exists for psychometrically strong measures in implementation science. Without them, the field cannot produce cumulative knowledge about implementation barriers, facilitators, or processes, or generate sound evidence about which implementation strategies work best, when, and for whom. The purpose of this study was to develop and test measures of constructs of the *Inner Setting* domain of the CFIR [9].

The “Inner Setting” of organizations has been identified as an important set of constructs that can influence the implementation of new research findings into practice [9]. There have been a number of useful definitions of the Inner Setting that help clarify its meaning and potential measurement. For example, Greenhalgh et al. developed a model to explain how innovations in health service delivery can diffuse through organizations; the authors described the organizational (inner) context which included both antecedents for innovation and readiness for innovation [10]. They also highlighted that organizations provide widely differing inner contexts for innovation implementation, and some characteristics of organizations (e.g., structure, culture) influence the likelihood that an innovation will be successfully adopted and incorporated into their usual practice. Lash et al. (2011) described the Inner Setting as the clinic or organizational context in which the intervention will exist [11]. Although we have seen an advance in the literature regarding conceptualization of the Inner Setting contexts and their influence on innovation adoption and implementation, empirical work to quantitatively measure the Inner Setting constructs is limited.

The CFIR was developed by reviewing and synthesizing constructs across 19 implementation and dissemination theories and frameworks into a unified typology [9]. The CFIR includes 37 constructs within 5 major domains: *Inner Setting*, *Outer Setting*, *Intervention Characteristics*, *Characteristics of Individuals*, and the *Process of Implementation*. The Inner Setting domain includes 5 constructs: *Structural Characteristics*, *Network and Communications*, *Culture*, *Implementation Climate*, and *Readiness for Implementation* [9], and another 9 sub-constructs (e.g., *Learning Climate* and *Available Resources*). While the framework describes these domains and constructs within them, it does not articulate relations between constructs or how they may interact to influence implementation. Accurate measurement is needed to begin to understand these relationships and to test whether individual or multiple constructs influence implementation.

This paper describes the work of the Cancer Prevention and Control Research Network (CPCRN) to develop measures for the Inner Setting domain of CFIR and assess the psychometric properties of those measures using data from a multi-state sample of FQHCs. The CPCRN is a group of collaborating centers funded by the Centers for Disease Control and Prevention (CDC) and the National Cancer Institute (NCI), through the Prevention Research Centers Program since 2002 [12, 13]. Each CPCRN center has regional networks of academic, public health, and community organizations that work together to further the dissemination and implementation of EBAs for cancer prevention and control [14]. This article is based on research carried out by the CPCRN FQHC Workgroup. The goal of the FQHC Workgroup was to advance the dissemination and implementation of evidence-based cancer prevention and control programs in FQHCs that provide primary care to underserved populations. Aligned with this goal was the aim to identify factors that influence the implementation of cancer control EBAs beginning with the development of validated measures of CFIR constructs. This study focuses on the development and testing of measures for 7 constructs related to the Inner Setting domain. Work to develop measures of other CFIR constructs is described elsewhere [15].

## Methods

### Development of measures for the Inner Setting constructs

The development of measures occurred in 4 phases: first, we identified constructs of interest and compiled existing measures for those constructs; second, we generated items for each construct of interest by adapting items from existing measures and developing new items to create a set of preliminary measures; third, we pilot-tested and refined the preliminary measures; and fourth, we conducted a validation study with the refined measures. Since our goal was to develop measures of constructs that could potentially be targets for implementation interventions and could be implemented feasibly within the FQHCs, we chose CFIR constructs that were relevant for FQHCs, modifiable, and hypothesized to be measurable with few items.

For all the steps described above, we used a consensus development process. We made decisions about what constructs to include at a CPCRN meeting that included CPCRN investigators and other implementation science experts. We discussed each Inner Setting construct and sub-construct and chose a preliminary set of constructs based on expert opinion about importance, changeability, and feasibility for measurement. Following the in-person meeting, the CPCRN FQHC Workgroup held two more in-person meetings and a series of teleconference discussions to make final decisions on the constructs and other development steps described above. We ultimately selected 15 out of 37 CFIR constructs to create measures for. Among these were 5 constructs that fall within the Inner Setting domain: Culture, Implementation Climate, Learning Climate, Leadership Engagement, and Available Resources*.* CPCRN sites then each took the lead on searching for items for one or more constructs, and the team held weekly meetings for several months and made decisions collectively about the items chosen as described below.

#### Identification and selection of items

We began our identification of the Inner Setting measures by drawing on existing surveys that had been administered in FQHCs. Specifically, we reviewed a survey created by the Association of Asian Pacific Community Health Organizations (AAPCHO) to study capacity for implementation of evidence-based interventions for cancer screening [16]. We chose the AAPCHO because it was highly related and allowed us to build on previous work. This survey included the Practice Adaptive Reserve (PAR) scale which had previously been used in the evaluation of the national Patient-Centered Medical Home Demonstration Project [17–19]. First, we identified items from the AAPCHO survey that matched CFIR constructs based on the construct definitions [9] and the face validity of items. We held multiple group discussions to reach consensus on the “match”. For constructs that did not have matching items from the AAPCHO survey or had items that did not fully reflect their definitions, we conducted a literature search for other existing measures. We started with models and frameworks included in the CFIR to see if they referred to measures of specific constructs. We also searched the following electronic databases: PubMed, CINAHL, ISI Web of Science, and PsycINFO for peer-reviewed articles published in the past 15 years to identify relevant measures. We used search terms such as CFIR, inner-setting, implementation culture, and other construct names to identify measures and constructs. In addition to the search, we also reviewed measures listed on the Grid Enabled Measures (GEM) and Society of Implementation Research Collaboration (SIRC) websites. We then compiled all the potential measures for those constructs and had extensive discussions to select items from each. We used the following criteria for item selection: (1) items fit the CFIR definition of the constructs, (2) items had been used in health related settings (e.g., public health, healthcare, mental health, and school) and were relevant for FQHCs or could be adapted to the FQHC setting, and (3) items fit the goals of the survey and were from published studies with measures that demonstrated some evidence of reliability (e.g., internal consistency) and validity (e.g., construct validity) in previous research.

In searching for *Culture* measures, we identified two sub-constructs not explicitly listed in the CFIR, *Stress* [20] and *Effort* [21], which were assessed separately. We decided to include these sub-constructs in addition to a more general measure of culture because the workgroup members believed that while related, these constructs were likely distinct. Therefore, our final list of the Inner Setting measures included 38 items to measure 7 constructs and sub-constructs: Culture Overall (CFIR construct; 9 items), Culture Stress (sub-construct based on the work of Patterson [21]; 4 items), Culture Effort (sub-construct based on the work of Lehman [20]; 5 items), Implementation Climate (CFIR construct; 4 items), Learning Climate (CFIR sub-construct; 4 items), Leadership Engagement (CFIR construct; 4 items), and Available Resources (CFIR sub-construct; 7 items). Definitions for each the Inner Setting construct and sub-construct are described in Table 1.

**Table 1:** The Inner Setting constructs, definitions, items, and sources

Construct name	Definition	Source	Items in the main survey
Sub-construct^a^			
Culture	Norms, values, and basic assumptions of a given organization	Practice Adaptive Reserve Scale (Jaen 2010) [19]	A03, A05, A07, A08, A09, A10, A22, A16, A21
Stress^b^	Perceived strain, stress, and role overload	TCU Organizational Readiness for Change (Lehman 2002) [20]	A36, A37, A38, A39
Effort^b^	How hard people in organizations work toward achieving goals	Organizational Climate Measure. (Patterson 2005) [21]	A40, A41, A42. A43, A44
Implementation climate-general	The shared receptivity of involved individuals to an intervention and the extent to which use of that intervention will be “rewarded, supported, and expected within their organization”	Community Clinical Oncology Program Survey (Weiner, 2011) [35]	C11^c^, C12^c^, C13^c^
		Implementation Climate Assessment (Klein, Conn, and Sorra 2001) [31]	C05^c^
Learning climate	A climate in which (a) leaders express their own fallibility and need for team members’ assistance and input; (b) team members feel that they are essential, valued, and knowledgeable partners in the change process; (c) individuals feel psychologically safe to try new methods; and (d) there is sufficient time and space for reflective thinking and evaluation (in general, not just in a single implementation)	Practice Adaptive Reserve (Jaen 2010) [19]	A01, A06,A15, A19
Readiness for implementation			
Tangible and immediate indicators of organizational commitment to its decision to implement an intervention, consisting of 3 sub-constructs. Implementation readiness is differentiated from implementation climate in the literature, by its inclusion of specific tangible and immediate indicators of organizational commitment to its decision to implement an intervention.			
Leadership engagement	Commitment, involvement, and accountability of leaders and managers	Practice Adaptive Reserve Scale (Jaen 2010) [19]	A11, A12, A13, A14.
Available resources	The level of resources dedicated for implementation and on-going operations including money, training, education, physical space, and time	ORCA (Helfrich 2009) [42]	A35a, A35b, A35c, C20a^c^, C20b^c^, C20c^c^, C20d^c^

^a^From CFIR except where noted

^b^Name and definition from the source of an item

^c^Items asked about a specific EBA

Item numbers correspond with the original survey

#### Item adaptation and survey development

The identification of measures made it clear that some constructs could be measured generally, that is, they did not necessarily need to be tied to a particular implementation effort or EBA, while others required specific anchoring about what EBA the item was referring to. Selected items were adapted for the context of improving colorectal cancer (CRC) screening in FQHCs. For intervention-specific constructs, such as Implementation Climate, items were also adapted to the specific EBA for CRC screening that the FQHC was implementing (captured in another section of the survey). EBA options were selected from those recommended by the Guide to Community Preventive Services (Community Guide) for increasing CRC screening (www.thecommunityguide.org).

Additionally, since we were interested in understanding factors influencing implementation of several EBAs for increasing CRC screening, participants were first asked about the level of implementation of each Community Guide recommended EBA and then asked questions related to CFIR constructs that were specific to the EBA being implemented. Because of constraints on the length of the survey, when a respondent indicated that the FQHC was implementing more than one EBA, subsequent questions on CFIR constructs referred to only one of the EBAs mentioned. The survey automatically inserted only one of the EBAs using the following prioritization: provider reminders first, followed by patient reminders, one-on-one education, and provider assessment and feedback. For example, if the clinic responded that they were implementing both provider reminders and one-on-one education, the follow-up questions would insert provider reminders. An example of a follow-up question is as follows: “the program is a top priority in the company” was an item to measure implementation climate by Klein et al. It was adapted as “Using <EBA> to increase CRC screening rates is a top priority in the clinic” in our measure. Depending on which EBAs were used by the clinic, as indicated by previous answers, the question appeared online with a specific EBA. Table 1 indicates whether an item was general or specific to an EBA.

#### Pilot testing and refinement

We programmed a web-based survey and then pilot-tested the survey in 4 FQHCs in 2 states (WA and TX). We also sought input from leaders at individual FQHCs and states’ Primary Care Associations (PCA) to ensure the appropriateness of the measures for FQHC clinic staff. More specifically, we asked leaders to review constructs for their importance and changeability as well as items for their understanding and representation of the constructs. We then held telephone meetings with leaders to discuss feedback. Feedback from leaders confirmed our selection of constructs and led to minor changes in the wording of some items.

### Recruitment and survey administration

We used a variety of strategies to recruit FQHCs to participate in the study [22]. While survey administration was customized, recruitment protocols were tailored based on the CPCRN existing partnerships with FQHCs in each participating state. Five CPCRN sites (WA, SC, TX, GA, CO) partnered with their state’s PCA. In 4 of these states (WA, TX, SC, CO), the PCA emailed their member FQHCs encouraging them to participate in the survey. Five CPCRN sites that had existing relationships with FQHCs (TX, GA, CA, CO, MO) invited them to participate in the survey by contacting them directly through email, telephone calls, or in-person meetings. One state PCA (SC) also directly recruited participants at a meeting of FQHC staff members.

In most cases, one individual from each participating FQHC was designated as the main contact, usually the clinic’s medical or administrative director. This individual was asked to complete questions about their clinic characteristics as well as send an introductory email with a link to the online FQHC CFIR survey to eligible staff members encouraging their participation. The online FQHC CFIR survey was programmed to allow a maximum of 10 staff from each clinic to complete the survey with a maximum of 3 providers (physicians, nurse practitioners, and physician assistants), 3 nurses or quality improvement staff, and 4 medical assistants (non-medical administrative staff were excluded). Between January 2013 and May 2013, providers and staff at FQHC clinics located in CA, CO, GA, MO, SC, TX, and WA completed the survey. Reminder emails were sent to potential participants at 2, 4, 6, and 8 weeks post-invitation. Incentives were offered to either individuals completing the survey or to FQHCs, whichever was preferred by the FQHC. If the clinic chose the individual incentive, participants received $25 gift cards. FQHCs that chose the clinic incentive received $250. One FQHC declined any incentives. All study procedures were approved by the Institutional Review Boards of each CPCRN Collaborating Center as well as the Coordinating Center at the University of North Carolina at Chapel Hill and the CDC.

### Data analyses

We assessed descriptive statistics for clinics which responded to the clinic characteristics survey (*n* = 52) and demographic information from FQHC CFIR survey respondents (*n* = 327). We also assessed descriptive statistics for FQHC CFIR survey measurement items. Since we collected data from individuals nested within clinics to measure clinic-level constructs, we used a series of confirmatory factor analysis (CFA) models to test factor structure. We first conducted single-level CFA models adjusting for the nested structure of the data for each of the following constructs: *Culture Overall*, *Culture Stress*, *Culture Effort*, *Implementation Climate*, *Learning Climate*, *Leadership Engagement*, and *Available Resources*. We used full information maximum likelihood estimation with robust standard errors to account for missing data and non-normality of survey items. We adjusted for the nested structure of the data by using the TYPE = COMPLEX command in Mplus. We used multiple indices to evaluate model fit as recommended by [23]: Chi square (non-significant value = good fit), comparative fit index (CFI, > 0.90 = adequate fit and > 0.95 = good fit), Tucker–Lewis Index (TLI, > 0.90 = adequate fit and > 0.95 = good fit), standardized root mean square residual (SRMR, < 0.08 = adequate fit and < 0.05 = good fit), and root mean square error of approximation (RMSEA, < 0.08 = adequate fit and < 0.05 = good fit) [23–26]. We considered model adjustments if modification indices revealed substantial model improvements that were theoretically meaningful (e.g., reverse-coded items or items that referred to a specific EBA versus a general EBA).

**Fig. 1:**
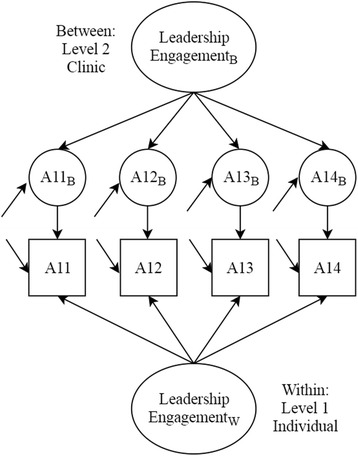
Example of multilevel confirmatory factor model for the Leadership Engagement Scale. The item number with B represents clinic-level items

We then conducted two sets of multilevel CFA models for each respective construct. Multilevel models allow for modeling the factor structure at the within-group or individual-level (level 1) and the between-group or the clinic-level (level 2), as illustrated in Fig. 1 [27, 28]. This approach allowed for testing whether the factor structure was similar at the individual-level and the clinic-level, which is assumed when only modeling individual data to represent a higher level. In the first set of multilevel models, we allowed factor loadings for both levels to freely estimate to test unrestricted models. We then tested a set of models where we constrained factor loadings to be equal across levels to determine if items were loading similarly for the individual (within-group) and clinic-levels (between-group). We compared model fit of constrained and unconstrained models between respective factors using Satorra-Bentler’s scaled chi square difference tests [29]. To assess fit for multilevel models, we used the same fit indices as previously listed, including the SRMR which is presented separately for the individual and clinic-levels for each model.

To evaluate internal consistency, we computed Cronbach’s alpha for each of the scales. We also examined discriminant validity by calculating correlation coefficients of each pair of scales using individual-level data and aggregated data by clinic (to represent the clinic-level). To further assess the reliability of mean scale scores aggregated at the clinic-level, we computed two intraclass correlation coefficients, ICC(1) and ICC(2), using one-way random effects ANOVA [30]. ICC(1) provides an estimate of the proportion of variance in a specific measure that is explained by group membership (FQHC clinic). The larger the value of ICC(1), the greater agreement or shared perception there is among raters within a group (FQHC clinic). ICC(2) indicates the reliability of the group-level mean scores. It varies as a function of ICC(1) and group size: the larger the value of ICC(1) and the larger the group size, the greater the value of ICC(2) and then, a more reliable group mean score. As recommended in the literature [30, 31], we used a threshold of 0.70 to indicate a reliable group score.

Finally, we tested an index of inter-rater agreement, the *r*_WG(*J*)_, to further assess the validity of clinic-level means as measures of clinic-level constructs. The *r*_WG(*J*)_ index indicates the degree of agreement among raters by comparing within-group variances to an expected variance under the null hypothesis of a distribution representing no agreement [32]. An *r*_WG(*J*)_ score above 0.70 indicates sufficient inter-rater agreement to compute FQHC clinic-level means for clinic-level constructs [33]. ICC(1), ICC(2), and *r*_WG(*J*)_ statistics at the clinic-level were computed for clinics with two or more respondents, so clinics with only one respondent were dropped from analyses. We used Mplus version 7.31 [34] for testing all CFA models. To test Cronbach’s alpha, correlation coefficients, ICC(1), ICC(2), and *r*_WG(*J*)_, we used SPSS version 23.

## Results

### Sample characteristics

A total of 327 individuals from 78 FQHCs responded to the survey. However, there were missing data across some survey questions and demographic variables (Tables 2 and 3). The majority of respondents were female (79%) and non-Hispanic individuals (64%) (Table 2). Thirty-seven percent were medical assistants, 36% were nurses, and 19% were physicians. Most participants had associate degrees or technical school diplomas (46%) or graduate or medical degrees (37%). Around 40% had worked at the clinic for 2 years or less, and 74% worked 40 h or more per week. Sixty percent of participants reported that they provided services in language(s) other than English.

**Table 2:** Characteristics of survey respondents (complete sample, *n* = 327)

	Total respondents	*N* (% of respondents)
Respondents’ characteristics		
Female	296	234 (79.0)
Ethnicity	296	
Non-Hispanic		189 (63.8)
Staff role	327	
Provider		63 (19.3)
Quality improvement/operations/clinic manager		28 (8.6)
Nurse		116 (35.5)
Medical assistant		120 (36.6)
Age (years)	296	
20–29		52 (17.6)
30–39		96 (32.4)
40–49		71 (24.0)
50 plus		77 (26.0)
Highest level of education completed	296	
High school or less/GED		13 (4.4)
Associates degree/some college or trade school		136 (45.9)
Bachelor’s degree		37 (12.5)
Graduate degree		110 (37.2)
Years employed at clinic	327	
0–2		129 (39.5)
3–4		52 (15.9)
5–9		71 (21.7)
≥ 10		75 (22.9)
Number of hours worked each week	327	
Less than 40 h		86 (26.3)
40 h		179 (54.7)
Greater than 40 h		62 (19.0)
Provide services in language(s) other than English	296	
Yes		177 (59.8)

**Table 3:** Means (standard deviations), ICCs, and standardized factor loadings (standard error) for level 1 and level 2

Item		*N*	*M* (SD)	ICC	Adjusted loadings	L1	L2
Culture							
A03	People at all levels openly talk about what is and isn’t working	327	3.66 (1.02)	0.15	0.668 (0.052)	0.666 (0.069)	0.728 (0.284)
A05	Most people in this clinic are willing to change how they do things in response to feedback from others	327	3.57 (0.98)	0.09	0.735 (0.036)	0.705 (0.048)	0.967 (0.092)
A16*	It is hard to get things to change in our clinic	322	3.11 (1.08)	0.10	0.521 (0.083)	0.452 (0.108)	0.994 (2.54)
A07	I can rely on the other people in this clinic to do their jobs well	323	3.62 (0.92)	0.10	0.671 (0.038)	0.626 (0.044)	0.983 (0.253)
A22	Most of the people who work in our clinic seem to enjoy their work	323	3.68 (0.86)	0.17	0.705 (0.046)	0.636 (0.051)	0.996 (0.177)
A08	Difficult problems are solved through face-to-face discussions	323	3.54 (0.96)	0.11	0.686 (0.048)	0.720 (0.044)	0.572 (0.418)
A09	We regularly take time to reflect on who we do things	323	3.64 (0.91)	0.11	0.723 (0.032)	0.731 (0.043)	0.992 (0.542)
A10	After trying something new, we take time to think about how it worked	323	3.65 (0.92)	0.10	0.729 (0.040)	0.777 (0.040)	0.870 (0.306)
A21	People in this clinic operate as a real team	322	3.57 (1.02)	0.15	0.762 (0.033)	0.700 (0.029)	0.997 (0.274)
Culture Stress							
A36	I am under too many pressures to do my job effectively	317	2.84 (1.08)	0.06	0.626 (0.087)	0.635 (0.102)	0.999 (0.001)
A37	Staff members often show signs of stress and strain	317	3.57 (0.99)	0.07	0.767 (0.041)	0.833 (0.038)	0.150 (12.36)
A38	The heavy workload here reduces program effectiveness	317	3.19 (1.03)	0.10	0.847 (0.072)	0.813 (0.042)	0.988 (1.573)
A39	Staff frustration is common here	317	3.44 (1.06)	0.12	0.765 (0.052)	0.859 (0.020)	−0.771 (4.552)
Culture Effort							
A40	People in this clinic always want to preform to the best of their abilities	317	4.02 (0.86)	0.12	0.695 (0.053)	0.637 (0.061)	0.999 (0.166)
A41	People are enthusiastic about their work	317	3.51 (0.83)	0.11	0.563 (0.060)	0.495 (0.061)	0.964 (0.123)
A42*	People in our clinic get by with doing as little as possible	317	3.57 (1.05)	0.24	0.546 (0.062)	0.434 (0.078)	0.916 (0.088)
A43	People are prepared to make a special effort to do a good job	317	3.85 (0.76)	0.18	0.858 (0.041)	0.839 (0.048)	0.981 (0.087)
A44*	People in this clinic do no put more effort into their work than they have to	317	3.38 (1.02)	0.12	0.560 (0.058)	0.500 (0.078)	0.896 (0.099)
Implementation Climate							
C11	Clinic staff are expected to help the <EBA> meet its goal (i.e., increase colorectal cancer screening rates)	259	3.85 (0.81)	0.28	0.535 (0.072)	0.473 (0.083)	0.754 (0.103)
C12	Clinic staff gets the support they need to implement <EBA>	259	3.40 (0.89)	0.20	0.879 (0.047)	0.844 (0.064)	0.982 (0.088)
C13	Clinic staff gets recognition for implementing <EBA> to increase colorectal cancer screening rates	259	2.98 (1.01)	0.09	0.708 (0.045)	0.685 (0.050)	0.992 (0.292)
C05	<EBA> to increase colorectal cancer screening rates is a top priority of the clinic	260	3.51 (0.91)	0.09	0.408 (0.088)	0.331 (0.091)	0.959 (0.491)
Learning Climate							
A01	We regularly take time to consider ways to improve how we do things	327	4.09 (0.88)	0.14	0.799 (0.045)	0.864 (0.030)	1.000 (0.000)
A02	People in our clinic actively seek new ways to improve how we do things	327	4.00 (0.89)	0.12	0.752 (0.043)	0.822 (0.040)	0.982 (0.082)
A06	This clinic encourages everyone to share ideas	327	3.86 (1.00)	0.17	0.829 (0.038)	0.722 (0.042)	0.946 (0.067)
A15	This clinic learns from its mistakes	322	3.71 (0.97)	0.16	0.600 (0.059)	0.501 (0.070)	0.804 (0.107)
A19	When we experience a problem in the clinic, we make a serious effort to figure out what’s really going on	322	3.86 (0.90)	0.13	0.610 (0.064)	0.530 (0.061)	0.908 (0.101)
Leadership Engagement							
A11	The clinic leadership makes sure that we have the time and space necessary to discuss changes to improve care	323	3.44 (1.12)	0.17	0.788 (0.033)	0.791 (0.038)	0.983 (0.039)
A12	Leadership in this clinic creates an environment where things can be accomplished	323	3.54 (1.08)	0.11	0.894 (0.020)	0.918 (0.019)	0.996 (0.108)
A13	Clinic leadership promotes an environment that is an enjoyable place to work	322	3.59 (1.06)	0.18	0.851 (0.034)	0.800 (0.042)	0.997 (0.062)
A14	Leadership strongly supports clinic change efforts	322	3.68 (1.01)	0.17	0.882 (0.023)	0.847 (0.029)	0.928 (0.067)
Available Resources							
A35a	In general, when there is agreement that change needs to happen in the clinic we have the necessary support in terms of: budget or financial resources	317	3.13 (1.02)	0.09	0.330 (0.072)	0.340 (0.080)	0.310 (0.156)
A35b	In general, when there is agreement that change needs to happen in the clinic we have the necessary support in terms of: training	317	3.39 (0.99)	0.13	0.421 (0.069)	0.424 (0.074)	0.407 (0.214)
A35c	In general, when there is agreement that change needs to happen in the clinic we have the necessary support in terms of: staffing	317	3.13 (1.04)	0.11	0.452 (0.067)	0.451 (0.068)	0.502 (0.263)
C20a	The following are available to make <EBA> work in our clinic: equipment and materials	258	3.67 (0.88)	0.12	0.698 (0.049)	0.690 (0.054)	0.882 (0.239)
C20b	The following are available to make <EBA> work in our clinic: patient awareness/need	258	3.56 (0.87)	0.04	0.805 (0.041)	0.803 (0.052)	0.857 (0.199)
C20c	The following are available to make <EBA> work in our clinic: provider buy-in	258	3.40 (0.90)	0.07	0.645 (0.073)	0.634 (0.076)	0.915 (0.558)
C20d	The following are available to make <EBA> work in our clinic: intervention team	258	3.16 (0.96)	0.10	0.711 (0.057)	0.699 (0.056)	0.947 (0.470)

Level 1 corresponds to individual and level 2 to clinic. Questions’ response options were 1—Strongly Disagree, 2—Disagree, 3—Neutral, 4—Agree, and 5—Strongly Agree

*ICC* intraclass correlation coefficient

*Indicates reverse-scored item

There was an average of about 4 respondents per clinic. Thirty-nine clinics had 1–3 respondents, 22 clinics had 4–6 respondents, and 17 clinics had 7–10 respondents. Of the 78 clinics, 19 were from WA, 15 from TX, 22 from CO, 10 from SC, 5 from GA, 6 from CA, and 1 from MO. A total of 52 clinics completed a separate clinic characteristics survey. Based on survey results from this subsample, the majority of the clinics (64%) served 5000 patients or more in 2012. Under half the clinics had ≥ 50% of patients uninsured and ≥ 40% of patients with limited English proficiency.

### Factorial validity

Item means ranged from 2.84 (± 1.08) to 4.09 (± 0.88) while item sample sizes ranged from 258 to 327 (Table 3). The majority of item response distributions were negatively skewed. With the exception of the Culture Stress model, fit for the Inner Setting constructs was good to excellent (RMSEA ≤ 0.08, CFI ≥ 0.95, TLI > 0.93, SRMR ≤ 0.04) (Table 4). The RMSEA value for the Culture Stress model indicated poor fit (> 0.08); however, the other indicators suggested good model fit. Almost all item factor loadings adjusted for the nested data structure were greater than 0.40 with the exception of item A35a in the Available Resources model (Table 3). Three models contained correlated residual variances: Culture Stress, Learning Climate, and Available Resources. Reasons for correlating residuals included reverse scored items and questions that were focused on a specific EBA versus more general resources within the same construct.

**Table 4:** Model fit (complex models)

Model	χ^2^	df	RMSEA	CFI	TLI	SRMR
Culture Overall	71.72	27	0.071	0.950	0.933	0.040
Culture Stress	11.40	2	0.122	0.974	0.921	0.033
Culture Effort^a^	0.85	4	0.000	1.000	1.025	0.006
Implementation Climate	2.34	2	0.026	0.998	0.993	0.023
Learning Climate^b^	12.35	4	0.080	0.985	0.963	0.027
Leadership Engagement	3.69	2	0.051	0.997	0.991	0.011
Available Resources^c^	13.22	11	0.025	0.995	0.990	0.025

^a^Correlated residual variance between A42 and A44

^b^Correlated residual variance between A19 and A15

^c^Correlated residual variance between A35a and A35b, A35a and A35c, A35b and A35c

Table 3 includes the variance explained by the clinic for each respective item. Results indicated the average ICC across all items was 0.13 with a range from 0.04–0.28. These results suggest that on average 13% of the variance for items was explained by the clinic, supporting the use of multilevel models [23]. Model results for the unconstrained multilevel CFA models were relatively consistent with results adjusted for clustering. The level 1 factor loadings were similar to the adjusted factor loadings while the level 2 factor loadings were consistently higher. Unconstrained models for Culture Effort and Available Resources demonstrated good model fit across all indices whereas Implementation Climate and Learning Climate had good model fit for most indices (Table 5). Unconstrained models for Culture Overall, Culture Stress, and Leadership Engagement had inconsistent fit results suggesting weaker (yet still good) fitting models relative to the other constructs.

**Table 5:** Model fit (two level)

Model	χ^2^	df	RMSEA	CFI	TLI	SRMR (*w*)	SRMR (*b*)
Culture Overall	216.25	54	0.096	0.891	0.854	0.047	0.105
Culture Overall Constrained	171.56	62	0.074	0.926	0.914	0.046	0.090
Culture Stress	18.73	5	0.093	0.978	0.947	0.034	0.683
Culture Stress Constrained	45.61	8	0.117	0.945	0.917	0.031	0.216
Culture Effort^a^	5.13	8	0.000	1.000	1.015	0.008	0.019
Culture Effort Constrained	17.08	12	0.037	0.989	0.982	0.020	0.098
Implementation Climate	14.28	4	0.099	0.957	0.870	0.033	0.054
Implementation Climate Constrained	18.91	7	0.081	0.950	0.914	0.033	0.086
Learning Climate^b^	33.31	9	0.091	0.970	0.933	0.033	0.034
Learning Climate Constrained	31.37	13	0.066	0.977	0.965	0.031	0.074
Leadership Engagement	41.19	4	0.170	0.959	0.878	0.019	0.032
Leadership Engagement Constrained	37.27	8	0.106	0.968	0.952	0.021	0.027
Available Resources^c^	52.05	22	0.066	0.956	0.916	0.029	0.103
Available Resources Constrained	49.60	28	0.049	0.968	0.952	0.033	0.202

^a^Correlated residual variance between A42 and A44

^b^Correlated residual variance between A19 and A15

^c^Correlated residual variance between A35a and A35b, A35a and A35c, A35b and A35c

When evaluating constrained models, the relative model fit appeared to improve for Culture Overall, Implementation Climate, Learning Climate, Leadership Engagement, and Available Resources (Table 5). Comparing constrained to unconstrained models using Satorra-Bentler’s scaled chi square difference tests revealed no significant differences in model fit. These results suggest factor loadings were similar for the within- and between-group portions of the model since allowing parameters to freely estimate did not significantly improve fit. Notably, the SRMR values were higher for the between-group portion of the model compared to the within-group portion suggesting the models fit the individual data better than the group-level data. Culture Stress had very high SRMR values for the between portion of both constrained and unconstrained models leading to insufficient support for use of this measure at the clinic-level (Table 5). Furthermore, the level 2 factor loadings of the Culture Stress model suggested an unexpected weak relation with item A37 and an inverse relation with item A39 (Table 3). Both factor loadings for these items were inconsistent with the level 1 and adjusted factor loadings, which were likely contributing to model misfit for the between portion of the model.

### Internal consistency

We estimated inter-item consistency of each of the 7 Inner Setting constructs. Overall, Cronbach’s alpha estimates were good (0.7 ≤ *α* < 0.9) or excellent (*α* ≥ 0.9) for all scales. Estimates were as follows: Culture Overall = 0.89, Culture Stress = 0.85, Culture Effort = 0.79, Available Resources = 0.81, Implementation Climate = 0.72, Learning Climate = 0.85, and Leadership Engagement = 0.92.

### Discriminant validity

We assessed discriminant validity by examining the correlations among constructs using the average score of each scale at the individual- and clinic-levels (Table 6). Three of the correlations, Culture Overall and Learning Climate, Culture Overall and Leadership Engagement, and Learning Climate and Leadership Engagement had values above 0.80 at both the individual and clinic-levels suggesting there may be some measurement overlap between constructs. The other correlations were well below the threshold, so good discriminant validity was shown across most the Inner Setting dimensions.

**Table 6:** Correlation coefficients for 7 dimensions of the Inner Setting

Scale	Available Resources	Culture Overall	Culture Stress	Culture Effort	Imp. Climate	Learning Climate	Leadership Engagement
Available Resources	1.00	0.61	− 0.41	0.44	0.73	0.49	0.57
Culture Overall	0.55	1.00	− 0.49	0.62	0.36	0.80	0.87
Culture Stress	− 0.40	− 0.45	1.00	− 0.47	− 0.30	− 0.26	− 0.39
Culture Effort	0.29	0.58	− 0.39	1.00	0.38	0.34	0.42
Imp. Climate	0.62	0.33	− 0.28	0.26	1.00	0.25	0.37
Learning Climate	0.53	0.83	− 0.38	0.40	0.32	1.00	0.81
Leadership Engagement	0.57	0.85	− 0.46	0.48	0.38	0.81	1.00

Correlations using average score for each scale; individual-level data are below the diagonal and clinic-level data are above the diagonal

### Inter-rater reliability and agreement statistics

Inter-rater reliability and inter-rater agreement statistics were computed to assess the reliability and validity of computing clinic-level means from the individual-level data. The results are presented in Table 7. With the exception of Culture Effort, the ICC(1) values of the scales were statistically significant and indicated that 10 to 22% of the variance in scale scores occurred between clinics. The ICC values for Culture Effort were negative suggesting there was a greater amount of variance within clinics versus between clinics for scale scores. When examining the ICC(2) values, none met the threshold of 0.80. Using the uniform distribution *r*_WG(*J*)_ values indicated good agreement for all 7 scales, ranging from 0.73 to 0.91. Using a slightly skewed distribution (and more conservative estimate) revealed *r*_WG(*J*)_ values ranging from 0.53–0.76 where only Culture Stress and Leadership Engagement demonstrated weaker agreement values below 0.70. These data support aggregation of clinic-level constructs for Culture Overall, Available Resources, Learning Climate, and Implementation Climate. However, there is weaker evidence supporting aggregation of Culture Stress, Culture Effort, and Leadership Engagement based on ICC and the more conservative *r*_WG(*J*)_ values.

**Table 7:** Clinic-level inter-rater reliability and agreement statistics

Scale	ICC(1)	ICC(2)	*r*_WG(*J*)_ Uniform distribution	*r*_WG(*J*)_ Slightly skewed distribution
Culture Overall	0.15*	0.42	0.91	0.76
Culture Stress	0.10*	0.32	0.73	0.53
Culture Effort	− 0.01*	− 0.05	0.88	0.74
Available Resources	0.12*	0.32	0.82	0.70
Learning Climate	0.21*	0.53	0.86	0.76
Implementation Climate	0.22*	0.50	0.86	0.72
Leadership Engagement	0.17*	0.46	0.78	0.61

Using average score for each scale

*p < 0.05

## Discussion

This study sought to identify, develop, and test measures that assess multiple dimensions of the CFIR Inner Setting domain. Our findings suggest that these measures exhibit adequate or good psychometric properties. More specifically, CFAs, inter-item consistencies, and correlation analyses indicated our Inner Setting measures have structural validity, reliability, and discriminant validity. Additionally, multilevel CFA results and inter-rater reliability and agreement analyses support using clinic-level means computed from individual data for most constructs.

Based on CFA results, scales with the strongest evidence for structural validity were Culture Effort and Available Resources. There was also moderate to strong evidence supporting the structural validity of Culture Overall, Implementation Climate, Learning Climate, and Leadership Engagement where the majority (but not all) of the fit indices suggested good or excellent fit. Culture Stress had the weakest evidence for structural validity, which could in part be due to the limited number of items (4) with one item focused on the individual (A36) whereas the other items were about the clinic (A37-A39).

When evaluating discriminant validity, constructs were differing from each other with the exception of Culture Overall, Learning Climate, and Leadership Engagement. We would expect there to be overlap given all these constructs are part of the Inner Setting. However, the stronger relation observed between these constructs is likely due to the fact that they can influence each other. For example, in this study, we included items that assessed the level of support the leader of an organization provides to create a productive and enjoyable environment where communication is valued [34]. Evidence shows that the culture and climate of an organization is highly influenced by its leadership [17]. Likewise, the organization’s learning climate, which in our study was measured with items that assessed the communication, observation, and reflection, and the desire to make things better can be seen as important elements that would make a clinic more “ready” for an implementation effort [19]. While these constructs were correlated, they can be assessed and targeted independently with implementation interventions.

In implementation science studies, the level of measurement is often a challenge because, while we may be interested in understanding how contextual factors influence adoption, implementation, and sustainment of EBAs, we typically measure these constructs by obtaining data from individuals within that organization [35]. In many cases, these contextual factors constitute subjective perceptions of organizational norms, culture, and readiness that must be assessed at the individual-level and could potentially vary from one person to another particularly among individuals with different types of roles (e.g., provider vs clinic manager). Nevertheless, it is likely that assessments from multiple individuals could provide a more accurate reflection of these organizational characteristics than by obtaining this information from one organizational representative alone.

In our study, we used two different approaches that have been used in previous studies to assess whether data collected from individuals can be used to represent the clinic. These included: (1) using multilevel models with equality constraints on corresponding factor loadings for the between and within portion of the models [36] and (2) testing reliability and agreement statistics for the individual-level data [37]. The results between the two methods were relatively consistent in supporting the use of clinic-level constructs with a few exceptions. Culture Stress had poor fit for the clinic-level portion of the multilevel model in addition to having weaker levels of agreement. Multilevel Culture Effort models demonstrated strong indicators of fit; however, assessing the ICC(1) for the individual data indicated there was more variance in the scale scores within clinics than between clinics. Overall, there is good evidence to support the use of the scales at the clinic-level with the exception of the Culture Stress and Culture Effort scales where more research may be necessary.

This study is the first to develop a set of quantitative measures assessing the Inner Setting constructs from CFIR for use in FQHCs. A limited number of studies have rigorously examined the CFIR Inner Setting measures [15, 38]; some studies have used qualitative approaches [9] while other studies have attempted to measure the CFIR Inner Setting constructs quantitatively [38]. Some studies have quantitatively assessed the extent to which providers perceive certain CFIR constructs as important in implementing a particular behavior [9, 39] but do not measure the construct explicitly. Other studies have used existing measures or subscales to assess some but not all the CFIR Inner Setting domains. For example, Ditty et al. examined constructs from the Inner Setting domain of CFIR and explored their association with the implementation of an evidence-based behavioral therapy [40]. Using a sequential mixed methods approach that included a survey followed by qualitative interviews, the authors explored the relation of selected the Inner Setting variables with implementation of dialectical behavior therapy among trained clinicians. While this study assessed cohesion and communication, team climate for innovation, and on-going supervision using existing scales, constructs from other domains such as Leadership Engagement and Available Resources were not assessed [40]. Acosta et al. (2013) included measures of coalition functioning, leadership, and incorporation of new practices as covariates in evaluating the Assets Getting to Outcomes intervention, an implementation intervention for implementing programs that employed a positive youth development approach to prevention [41].

None of the published work, however, provides a way to measure the multiple dimensions of the Inner Setting domain. Emmons et al. expressed the need for developing and evaluating measures to assess the multidimensionality of organizational-level (inner setting) constructs [5]. Weiner et al. also highlighted that a robust measure could be a valuable diagnostic tool to guide implementation efforts in practice settings [7]. For example, stakeholders in clinical settings (and potentially other organizations) could use such a tool assess the level of culture, implementation climate, or other constructs from the Inner Setting. This information could inform the development or selection of implementation strategies to improve these, if assessments reveal deficits in any areas. Addressing these factors could lead to more efficient and effective implementation efforts in practice settings. Additionally, the measures could be used to assess change in these constructs over time. This study addressed both calls in the literature and requests from the practice communities by developing a psychometrically robust instrument useful for both research and practice.

This study has several strengths. To our best knowledge, this work is the first to develop quantitative measures of *Inner Setting*, based on the CFIR, for use in FQHCs. In addition, because of the focus on developing pragmatic measures that could be used in implementation research in FQHCs as well as by FQHCs themselves, we chose the Inner Setting constructs that were relevant to FQHCs, amenable to intervention change, and could be assessed with few items. Another strength of this study is that we used a rigorous scale development approach to assess the psychometric properties of our measures. This approach tested different forms of reliability and validity in addition to using multilevel CFA models to account for the individual and clinic-level aspects of the data. Lastly, this study was conducted in 78 clinics across 7 states, which represents a geographically diverse sample and strengthening the generalizability of results. However, more research needs to be done to test if the measures are valid in other settings and topic areas.

This study also has some limitations. The stage of implementation of EBAs could have influenced the measurement of some of the variables assessed. Another limitation was the varying numbers of respondents per clinic, with some clinics having as few as 3 respondents. Another potential limitation is that individual respondents played a variety of roles in patient care. These roles may influence their perception of certain clinic the Inner Setting characteristics and could also influence their perception of the extent to which an EBA is being implemented. Nevertheless, one would expect that even if particular clinic providers or staff may not be directly involved in the implementation, they would be able to assess (from their perspective) to what extent the program was being implemented. If they were not even aware of the program, they would likely indicate the program was not yet “fully implemented”. Lastly, while the CFIR builds on literature from studies conducted in many countries [10], many of the measures we drew from and the data we collected for the validation occurred within the USA. Therefore, although the broader constructs are likely applicable beyond North America, the specific measures described here may represent a cultural bias. Additional research is needed that would further validate these measures in other countries and languages.

## Conclusions

This study provides evidence that the Inner Setting measures described here have structural validity, reliability, and discriminant validity, and that they can be used to represent the clinic-level. Our findings also suggest the Inner Setting measures can be aggregated to represent the clinic-level. Measurement is crucial for any field, and our understanding of how contextual factors influence implementation as well as our ability to intervene upon these factors is dependent on our ability to measure them. This study provides information and measurement tools that can greatly contribute to research aimed at better understanding the implementation of evidence-based programs and practices in FQHC settings. It can also inform the development of implementation interventions to accelerate and improve the use of healthcare innovations, practices, and programs that will lead to increases in health and quality of life and decreased health disparities.

## Abbreviations

AAPCHO: Association of Asian Pacific Community Health Organizations
CDC: Centers for Disease Control and Prevention
CFA: Confirmatory factor analysis
CFI: Comparative fit index
CFIR: Consolidated Framework for Implementation Research
CPCRN: Cancer Prevention and Control Research Network
CRC: Colorectal cancer
EBA: Evidence-based approach
FQHC: Federally Qualified Health Center
ICC: Intraclass correlation coefficient
NCI: National Cancer Institute
PAR: Practice Adaptive Reserve
PCA: Primary Care Associations
RMSEA: Root mean square error of approximation
SRMR: Standardized root mean square residual
TLI: Tucker–Lewis Index
